# Exploring the effects of genetic variation on gene regulation in cancer in the context of 3D genome structure

**DOI:** 10.1186/s12863-021-01021-x

**Published:** 2022-02-17

**Authors:** Noha Osman, Abd-El-Monsif Shawky, Michal Brylinski

**Affiliations:** 1 Department of Biological Sciences, Louisiana State University, Baton Rouge, LA 70803 USA; 2 Department of Cell Biology, National Research Centre, Giza, 12622 Egypt; 3 Department of Medicine, Baylor College of Medicine, Houston, Texas 77030 USA; 4 Center for Computation and Technology, Louisiana State University, Baton Rouge, LA 70803 USA

**Keywords:** 3D genome structure, Genetic variation, Single-nucleotide polymorphism, Gene regulation, Chromosome conformation capture, Genome-wide association study, Topologically associating domains, Transcription factors, Enhancers, Breast cancer, Prostate cancer

## Abstract

**Background:**

Numerous genome-wide association studies (GWAS) conducted to date revealed genetic variants associated with various diseases, including breast and prostate cancers. Despite the availability of these large-scale data, relatively few variants have been functionally characterized, mainly because the majority of single-nucleotide polymorphisms (SNPs) map to the non-coding regions of the human genome. The functional characterization of these non-coding variants and the identification of their target genes remain challenging.

**Results:**

In this communication, we explore the potential functional mechanisms of non-coding SNPs by integrating GWAS with the high-resolution chromosome conformation capture (Hi-C) data for breast and prostate cancers. We show that more genetic variants map to regulatory elements through the 3D genome structure than the 1D linear genome lacking physical chromatin interactions. Importantly, the association of enhancers, transcription factors, and their target genes with breast and prostate cancers tends to be higher when these regulatory elements are mapped to high-risk SNPs through spatial interactions compared to simply using a linear proximity. Finally, we demonstrate that topologically associating domains (TADs) carrying high-risk SNPs also contain gene regulatory elements whose association with cancer is generally higher than those belonging to control TADs containing no high-risk variants.

**Conclusions:**

Our results suggest that many SNPs may contribute to the cancer development by affecting the expression of certain tumor-related genes through long-range chromatin interactions with gene regulatory elements. Integrating large-scale genetic datasets with the 3D genome structure offers an attractive and unique approach to systematically investigate the functional mechanisms of genetic variants in disease risk and progression.

**Supplementary Information:**

The online version contains supplementary material available at 10.1186/s12863-021-01021-x.

## Background

Cancer is a complex disease involving strong interactions between genetic and environmental factors, and the second leading cause of death globally [1, 2]. The dysregulation of oncogenes and/or tumor suppressor genes has an impact on cell proliferation and apoptosis in cancer pathogenesis through genetic alterations such as mutations [3, 4]. Further, the chromatin structure and regulatory elements can dysregulate gene expression subsequently leading to cancer development [5, 6]. Among different types of tumors, breast and prostate cancers create significant health problems worldwide because of their high incidence, health-related costs, and mortality rates [7, 8]. Breast cancer is the most predominant malignancy in women with a high incidence rate, prevalence, and mortality [9–11]. The extremely complex and heterogenous etiology of breast cancer, involving numerous components such as endocrine and environmental factors, other medical conditions, and genetic susceptibility [12, 13], is not yet fully understood. Prostate cancer is the second most frequent tumor in men worldwide [14]. Similar to breast cancer, it has a high genetic heritability with ethnic and geographical factors having a significant effect on the disease progression as well [15].

Genome-wide association study (GWAS) provides a systematic way to identify genetic risk factors for various diseases, including cancer [16], type 2 diabetes [17], Alzheimer’s disease [18], inflammatory bowel disease [19], and many others. The goal of GWAS is to reveal genotype-phenotype associations by detecting genomic loci that are common and low-penetrant in a specific disease state without any prior knowledge of their locations and functions [20, 21]. In the last decade, GWAS conducted on many different tumor types, including pancreatic [22], ovarian [23], lung [24], prostate [25], and breast cancer [26], identified numerous risk alleles, most of which are common and individually confer only a modest increase in disease risk. For instance, GWAS revealed 31 novel genetic susceptibility loci associated with the genetic predisposition for breast cancer [27] and 12 novel loci for prostate cancer [28]. Notably, the vast majority of genetic variants identified through GWAS (> 90%) are located in the non-coding regions of the genome [29]. These variants can provide useful insights into mechanisms responsible for the development and progression of various diseases through the alteration of regulatory elements, such as transcription factors (TFs) and active enhancers, affecting the expression of certain disease-related genes [30, 31].

A number of studies investigated the downstream effects of a single-nucleotide polymorphism (SNP) in disease states [32–35]. One of the first reports was focused on a single nucleotide substitution in the promoter region of β-thalassemic globin gene decreasing the expression of β-globin in patients with thalassemia [36]. Other studies investigated the effect of SNPs located in a promoter region on the promoter activity [37] as well as those located at TF binding sites affecting the binding of TFs and, subsequently, altering the gene expression [38]. Although the presence of SNPs in the non-coding regions of the genome, such as introns and intergenic regions, can alter the susceptibility to disease, the exact regulatory mechanisms of gene expression are often not fully elucidated [39, 40]. This difficulty can be attributed to the fact that SNPs may affect the expression of genes located even hundreds of kilobase pairs (kbp) away complicating the illumination of *cis*-regulatory mechanisms [31, 41]. Deciphering the effects of high-risk SNPs is not only critical to understand the molecular pathogenesis of cancer, but it can also improve cancer diagnostics and prognosis [42], and reveal potentially novel targets for pharmacotherapy [43].

Traditionally, the genome has intensively been studied as a unidimensional, linear entity often using the number of base pairs as a distance between various genomic elements. More recently, the three-dimensional (3D) structure of the genome started drawing significant attention because the regulation of gene expression and, consequently, cellular functions in physiology and disease cannot be grasped without considering the genome organization and the nuclear architecture. High-resolution chromosome conformation capture (Hi-C) is the latest variant of chromosome confirmation capture (3C) techniques developed to investigate the 3D genome structure using next-generation sequencing strategies [44]. This method enables researchers to profile pair-read contacts in all-versus-all manner in order to calculate the interaction frequency both within chromosomes (intra-chromosomal contacts) and between different chromosomes (inter-chromosomal contacts). The Hi-C resolution is determined based on the fragmentation of chromosomes and can vary from a low resolution of 1000 kbp to as high as 5 kbp, in which each fragment comprises 5000 base pairs [45]. Further, the genome is systematically arranged into topologically associating domains (TADs) defined as those genomic regions forming numerous self-interactions whose frequency is much higher compared to contacts with other parts of the same chromosome [46, 47]. TADs often contain multiple genes and regulatory elements, and have been shown to play a crucial role in the development of a wide array of diseases including cancer [48, 49]. Overall, the Hi-C data give invaluable insights into the 3D genome structure facilitating the identification of physical interactions among genetic variants, regulatory elements, and their corresponding target genes.

In situ Hi-C [50] was recently combined with whole-genome bisulfite sequencing at base resolution [51] to simultaneously profile chromatin conformation and DNA methylation in single cells [52]. Interestingly, this study revealed not only the coordinated DNA methylation status between distal genomic segments located in a spatial proximity in the nucleus, but also the heterogeneity of the chromatin architecture and the DNA methylome in a mixed population of cells. Integrating Hi-C with DNA methylation detection opens up a possibility to simultaneously characterize the cell-type-specific chromatin organization and epigenome in complex tissues. Other studies investigated the genetic variation related to human diseases in the context of the 3D genome structure assembled from the Hi-C data [53, 54]. For example, the Hi-C data have been collected for different cancer types in order to gain new insights into the effects of SNPs on regulatory elements leading to tumor progression. This approach can help identify high-risk mutations modulating gene expression in cancer by affecting regulatory elements located far away from their target genes in the linear genome [55–57].

The Hi-C data are often used in conjunction with the expression quantitative trait loci (eQTL) analysis to reveal risk loci contributing to cancer progression. For instance, the rs2981579 variant maps to the transcription start site of fibroblast growth factor receptor 2 (FGFR2) forming interaction peaks with several distal fragments [58]. These regions are located hundreds kbp from the capture region and co-localize with DNAse I hypersensitive sites, CTCF, FOXA1, GATA3, and ERα binding sites in breast cancer and normal breast epithelial cell lines. Translating these interactions helped explain the association of this SNP with FGFR2 gene regulation in breast cancer. Another example is rs4442975 associated with the susceptibility to breast cancer. According to the Hi-C data, this variant is located near a transcriptional enhancer forming physical interactions with the promoter region of insulin-like growth factor binding protein 5 (IGFBP5) [59]. IGFBP5 displays allele-specific gene expression with *g*-allele downregulating the expression of IGFBP5 leading to the increased susceptibility to breast cancer. Genetic variants are also associated with prostate cancer through long-range chromatin interactions with regulatory elements, such as the promoter regions of a specific gene (rs10486567) [54] and active enhancers regulating the expression of multiple disease-related genes (rs55958994) [60].

Although various studies were conducted to illuminate the effects of a specific genetic variation through chromatin interactions with selected gene regulatory elements, functional relationships among SNPs, regulatory elements, and disease-associated genes have not been systematically evaluated at the level of the entire human genome. In this communication, we present a large-scale analysis of the Hi-C data in the context of relationships among high-risk SNPs identified by GWAS for breast and prostate cancers, regulatory elements including TFs and enhancers, and genes associated with both diseases. The results highlight the importance of including the 3D genome structure in the investigation of the effects of genetic variation on gene regulation in cancer.

## Results

### Mapping genetic variants to regulatory elements and target genes

**Fig. 1:**
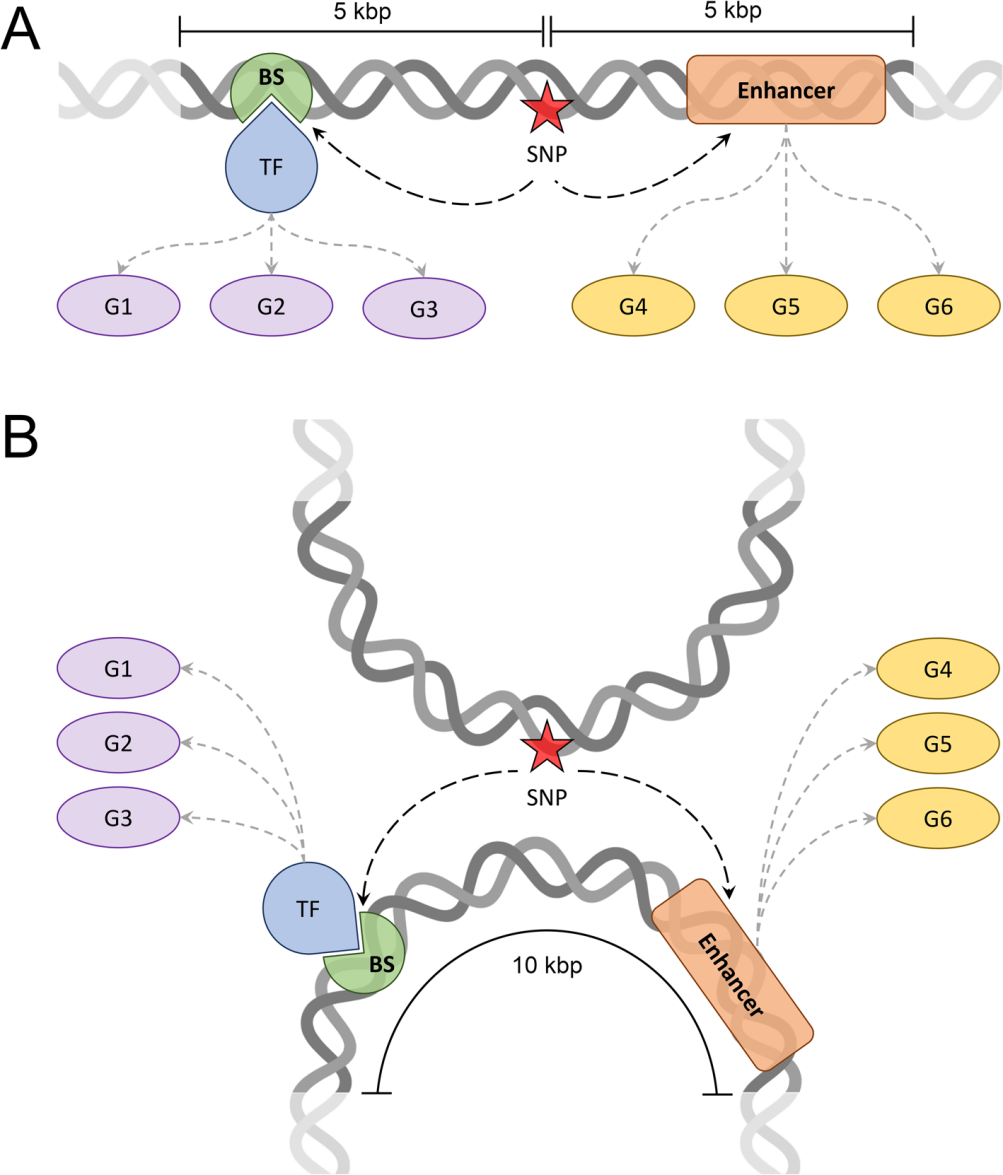
Schematic representation of the procedure to map SNPs to regulatory elements and target genes. The mapping is shown for **A** the 1D linear genome and **B** the 3D genome structure constructed at the Hi-C resolution of 10 kbp. Red stars are a SNPs highly associated with a disease at a *p*-value of ≤5 × 10^− 8^. Regulatory elements include transcription factors (TF, blue teardrops) and their binding sites (BS, green sectors), and enhancers (orange rounded rectangles). Each regulatory element is linked to its target genes (G1–3, purple ovals for TFs and G4–6, yellow ovals for enhancers). In (**A**), regulatory elements are identified within a DNA window of 10 kbp centered on the SNP, whereas in (**B**), regulatory elements are identified in a DNA fragment forming a physical contact with the fragment containing the SNP

In this study, we compare two distinct approaches to link genetic variants highly associated with disease, with a *p*-value of ≤5 × 10^− 8^ according to the GWAS data, to regulatory elements and their target genes (Fig. 1). The first approach, schematically shown in Fig. 1A, employs the unidimensional (1D) genome structure to identify those enhancers and TF binding sites located in the linear proximity up and downstream of a SNP. In this example, a TF binding site (green shape) is found downstream from a SNP (red star) and an enhancer (orange rectangle) is found upstream. For prostate cancer, the search distance is set to 5 kbp on both sides of the SNP in order to create a SNP-centered window whose size is the same as the resolution of the Hi-C data (10 kbp). Since the resolution of the Hi-C data for breast cancer used in this study is 5 kbp, we search for regulatory elements located 2.5 kbp down and 2.5 kbp upstream of a SNP. Both regulatory elements shown in Fig. 1A affect the expression of their target genes, either indirectly through TF (blue teardrop) binding (genes G1–3, purple ovals) or directly (genes G4–6, yellow ovals). The search for regulatory elements in the linear proximity from 808 SNPs highly associated with breast cancer identified 12 TF binding sites affecting 8 genes for 59 SNPs and 51 enhancers affecting 33 genes for 50 SNPs. A similar search conducted for 13,447 SNPs highly associated with prostate cancer resulted in 247 TF binding sites affecting 180 genes for 986 SNPs and 3851 enhancers affecting 613 genes for 7641 SNPs.

The second approach maps SNPs highly associated with cancer at a *p*-value of ≤5 × 10^− 8^ to regulatory elements located in the spatial proximity according to the 3D genome structure. Here, we utilize highly confident intra- and inter-chromosomal contacts obtained from the Hi-C data with a *q*-value of ≤0.05. Specifically, for each SNP, we collected those DNA fragments containing at least one regulatory element and forming physical contacts with that SNP. Next, we selected one fragment with the lowest *q*-value for a contact; in case of multiple fragments having the same lowest *q*-value for contacts, the longest-range fragment from the SNP was picked. As shown in Fig. 1B, a DNA fragment containing a disease-associated SNP (red star) physically interacts with another fragment through a highly confident long-range contact. In this example, the interacting fragment contains a binding site (green shape) for a TF (blue teardrop) and an active enhancer (orange rectangle). Just as in the first approach utilizing the 1D linear genome, these elements regulate the expression of their target genes, G1–3 (purple ovals) and G4–6 (yellow ovals), respectively.

Following this procedure, we identified 19,240 chromatin fragments forming highly confident contacts with 808 SNPs associated with breast cancer. Selecting only one long-range chromatin contact per SNP with the lowest *q*-value resulted in 702 fragments containing regulatory elements. These elements include 239 enhancers having 147 target genes in contact with 702 SNPs and 83 binding sites for TFs having 70 target genes in contact with 459 SNPs. Similar to breast cancer, selecting one long-range chromatin contact per SNP with the lowest *q*-value from 1,952,907 chromatin fragments forming contacts with 13,447 SNPs associated with prostate cancer resulted in 13,429 contacts. Among these interactions, 13,410 contacts are between 13,410 SNPs and 3585 enhancers with 747 target genes, and 1387 contacts are between 1387 SNPs and 324 binding sites for TFs with 190 target genes.

### Disease association of enhancers connected to genetic variants in cancer

**Fig. 2:**
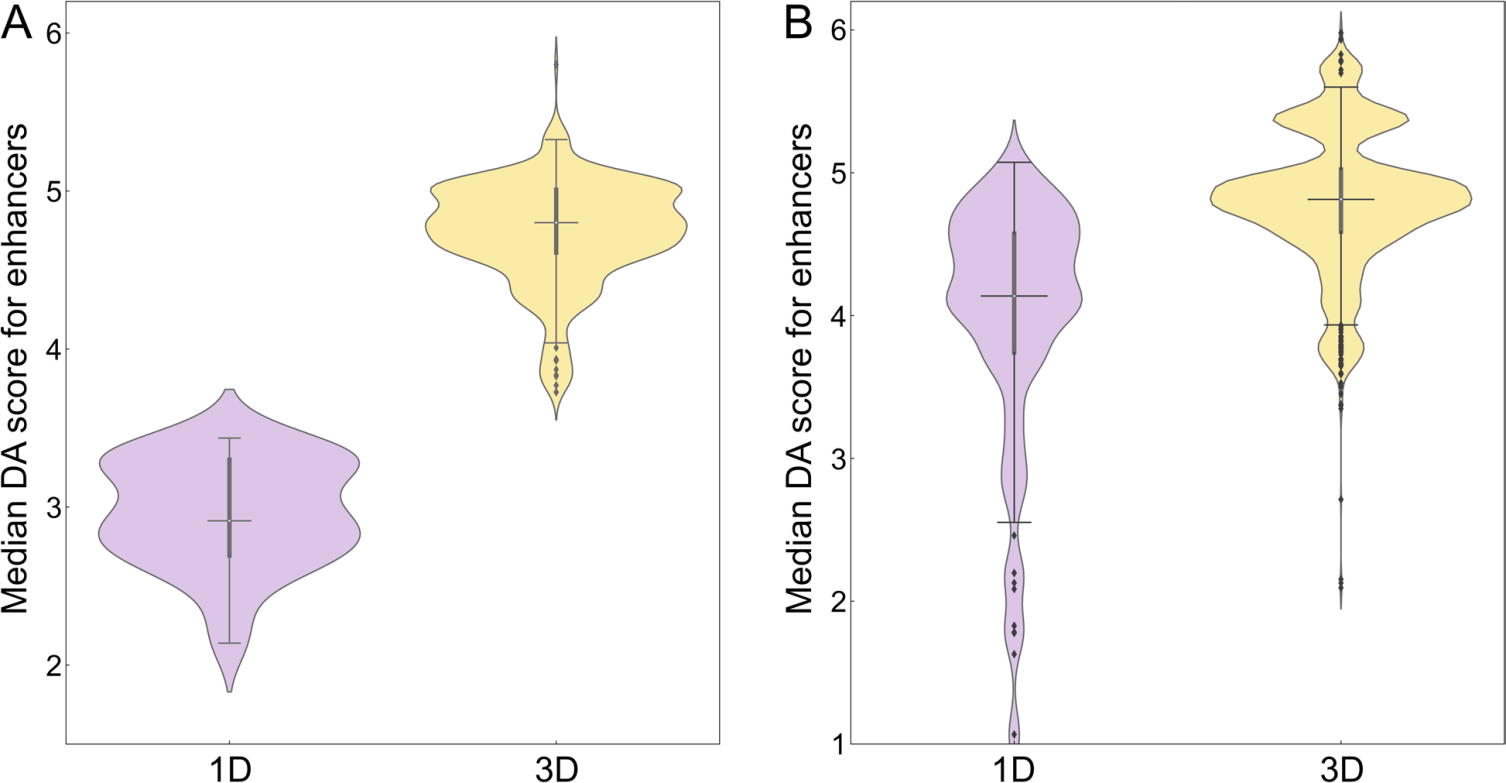
Distribution of disease-association (DA) scores for enhancers linked to SNPs. Enhancers were identified by mapping SNPs highly associated with **A** breast and **B** prostate cancer in the unidimensional (1D, purple violins) and the three-dimensional (3D, yellow violins) genome structure. In each violin, the horizontal black line is the median, the narrow gray box shows the first and third quantiles, and whiskers mark the minimum and maximum values excluding outliers, which are represented by black diamonds

**Fig. 3:**
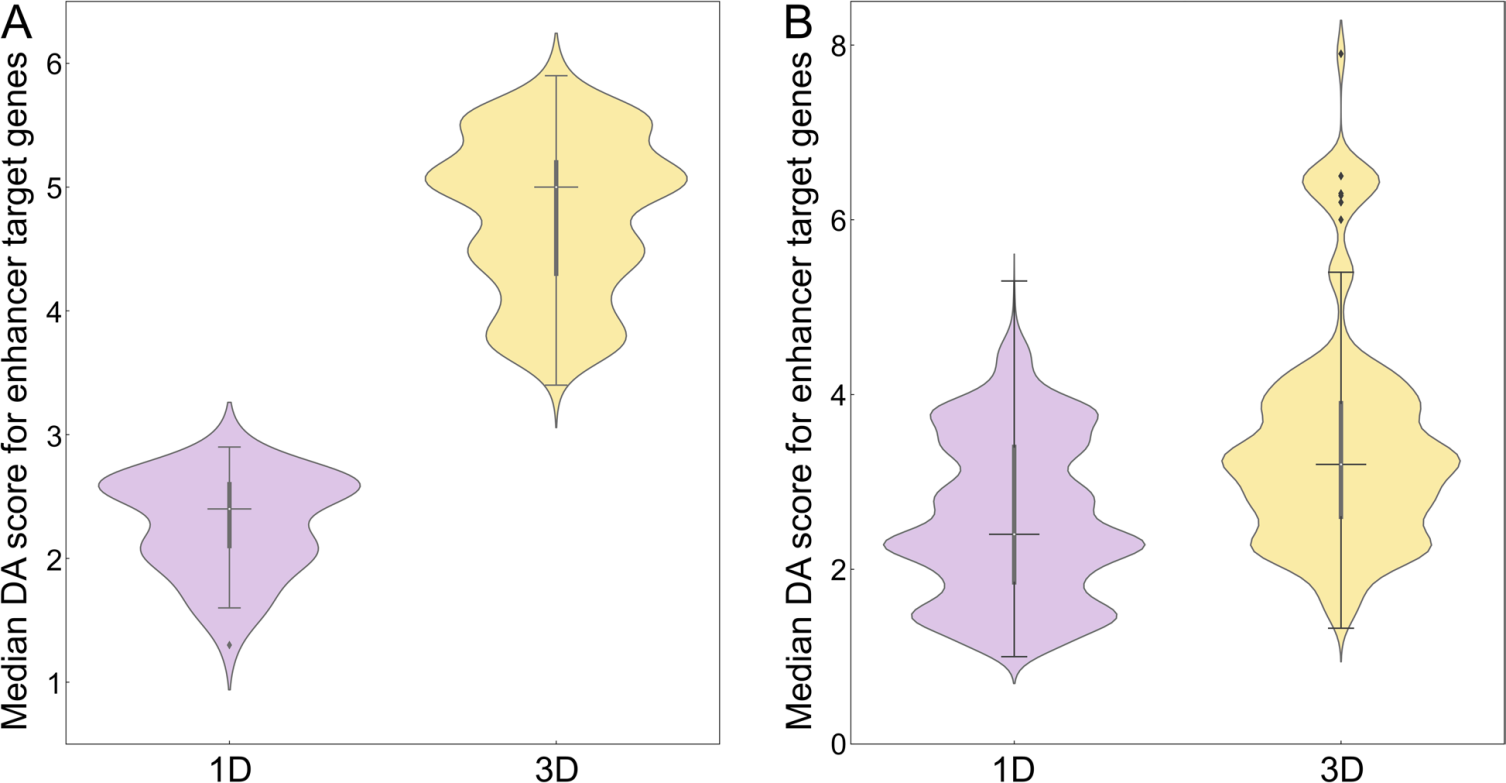
Distribution of disease-association (DA) scores for the target genes of enhancers linked to SNPs. Enhancers were first identified by mapping SNPs highly associated with **A** breast and **B** prostate cancer in the unidimensional (1D, purple violins) and the three-dimensional (3D, yellow violins) genome structure, and then linked to their target genes. In each violin, the horizontal black line is the median, the narrow gray box shows the first and third quantiles, and whiskers mark the minimum and maximum values excluding outliers, which are represented by black diamonds

In order to measure the relevance of those regulatory elements affected by SNPs to a disease, a series of disease association (DA) scores were computed. For each high-risk SNP, we calculated the median DA score for mapped enhancers and TFs as well as the median DA score for target genes whose expression is regulated by these elements. The number of SNPs along with quantile and inter-quantile range (IQR) values are reported in Table 1 (enhancers) and Table 2 (TFs). The distribution of DA scores for active enhancers is presented in Fig. 2. Figure 2A shows that the median (2nd quantile) DA score of 4.80 for enhancers linked to highly associated SNPs in breast cancer in the 3D genome structure is higher compared to 2.91 in the 1D linear genome (Table 1). Similar to breast cancer, Fig. 2B and Table 1 show that the median DA score for enhancers linked to SNPs highly associated with prostate cancer is higher in 3D (4.81) than 1D (4.14). To further corroborate these results, we computed DA scores for target genes whose expression is affected by enhancers linked to genetic variants in cancer. Figure 3 and Table 1 show that the median DA scores are systematically higher in 3D compared to 1D in breast cancer (Fig. 3A, 2.4 for 1D and 5.0 for 3D) and in prostate cancer (Fig. 3B, 2.4 for 1D and 3.2 for 3D). In addition to higher DA scores, IQRs are typically smaller in the 3D genome structure compared to 1D (Table 1), for instance, the IQR for the enhancer DA score is 0.40 in 3D and 0.61 in 1D for breast cancer, and 0.43 in 3D and 0.83 in 1D for prostate cancer.

**Table 1:** Disease association (DA) statistics for enhancers linked to significant SNPs in breast and prostate cancer. Statistics for enhancers identified with 1D and 3D approaches include the number of SNPs used in the analysis, quantiles, and the inter-quantile range (IQR). For each cancer type, DA scores for enhancers and their target genes are reported

Statistic	Breast cancer				Prostate cancer			
	Enhancer DA score		DA score for target gene		Enhancer DA score		DA score for target gene	
	1D	3D	1D	3D	1D	3D	1D	3D
# of SNPs	50	702	50	662	7,641	13,410	7,213	12,010
1^st^ quantile	2.69	4.61	2.1	4.3	3.74	4.59	1.9	2.6
2^nd^ quantile	2.91	4.80	2.4	5.0	4.14	4.81	2.4	3.2
3^rd^ quantile	3.30	5.01	2.6	5.2	4.57	5.02	3.4	3.9
IQR	0.61	0.40	0.5	0.9	0.83	0.43	1.5	1.3

**Table 2:** Disease association (DA) statistics for transcription factors (TFs) linked to significant SNPs in breast and prostate cancer. Statistics for enhancers identified with 1D and 3D approaches include the number of SNPs used in the analysis, quantiles, and the inter-quantile range (IQR). For each cancer type, DA scores for enhancers and their target genes are reported

Statistic	Breast cancer				Prostate cancer			
	TF DA score		DA score for target gene		Fraction of DA-TFs^**a**^		DA score for target gene	
	1D	3D	1D	3D	1D	3D	1D	3D
# of SNPs	59	459	42	210	986	1,387	759	1,387
1^st^ quantile	2.0	10.0	2.5	4.3	0.31	0.65	1.4	3.0
2^nd^ quantile	3.0	12.0	2.7	4.7	0.33	0.67	1.9	3.2
3^rd^ quantile	4.0	12.0	3.0	4.9	0.34	0.69	3.0	3.7
IQR	2.0	2.0	0.5	0.6	0.03	0.04	1.6	0.7

^a^ Fraction of disease-associated TFs within a set of all TFs linked to significant SNPs

### Disease association of transcription factors connected to genetic variants in cancer

**Fig. 4:**
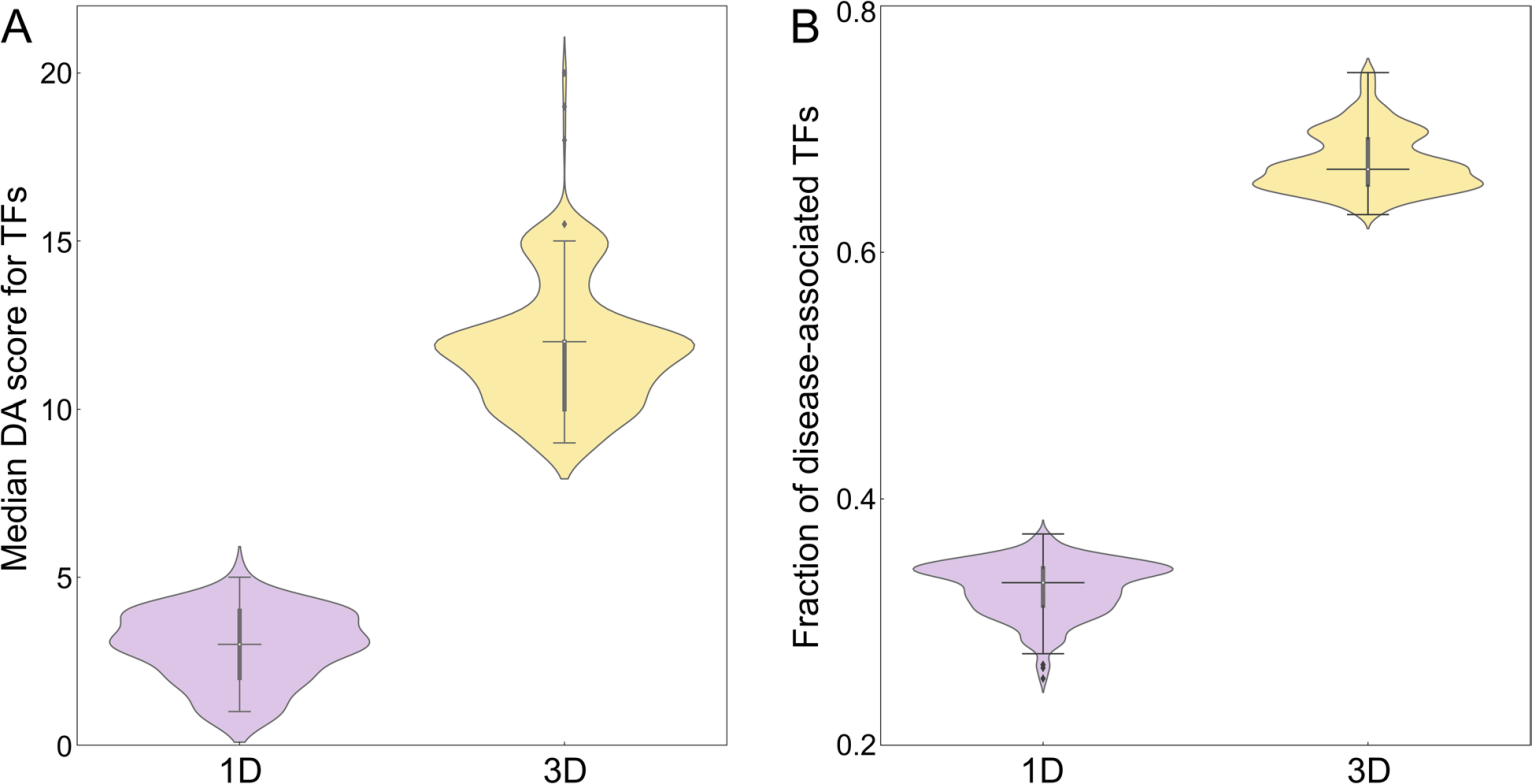
Analysis of the disease association of transcription factors (TFs) linked to SNPs. **A** The distribution of disease-association (DA) scores for TFs linked to SNPs highly associated with breast cancer. **B** The fraction of disease-associated TFs linked to SNPs highly associated with prostate cancer. TFs were identified by mapping SNPs in the unidimensional (1D, purple violins) and the three-dimensional (3D, yellow violins) genome structure. In each violin, the horizontal black line is the median, the narrow gray box shows the first and third quantiles, and whiskers mark the minimum and maximum values excluding outliers, which are represented by black diamonds

**Fig. 5:**
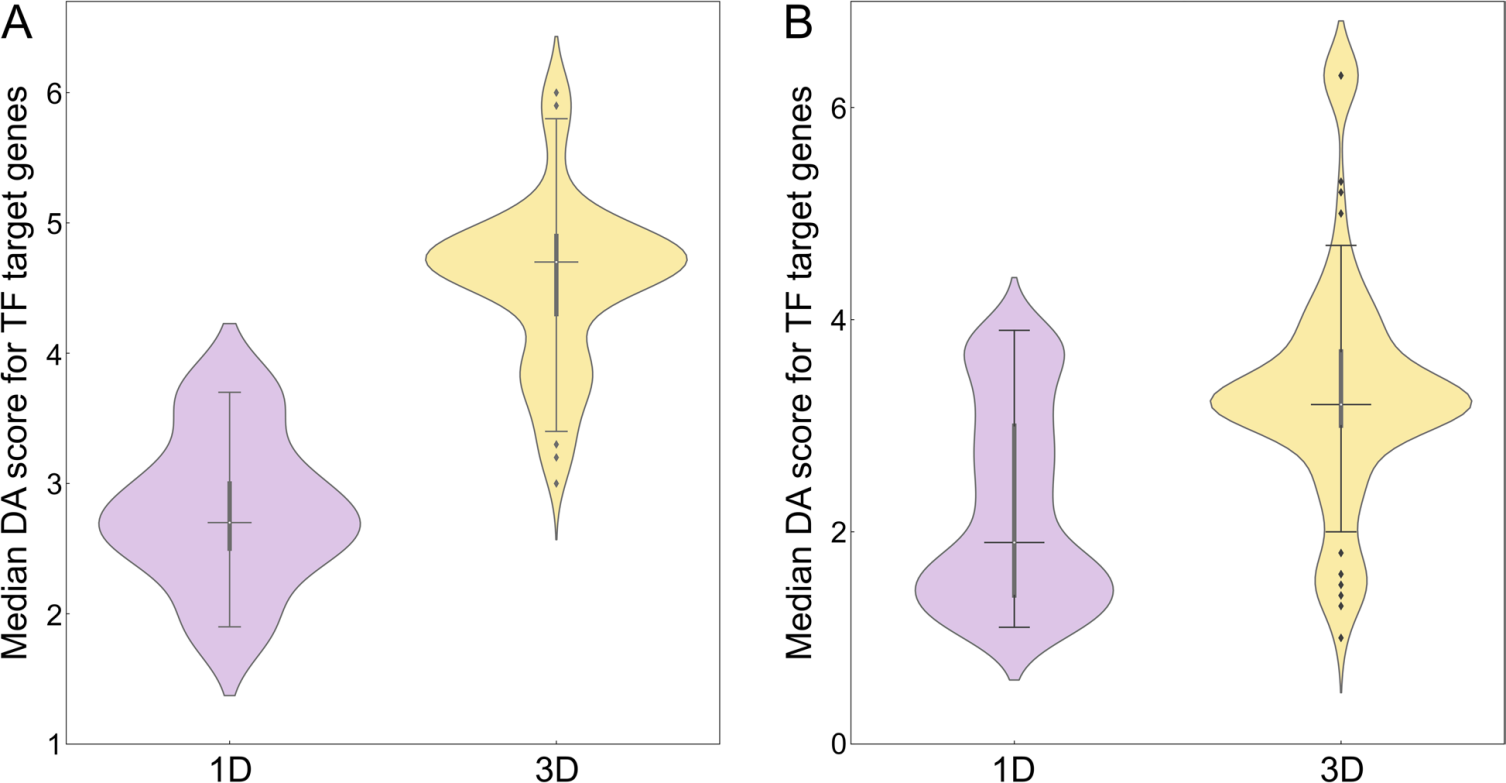
Distribution of disease-association (DA) scores for the target genes of transcription factors (TFs) linked to SNPs. TFs were first identified by mapping SNPs highly associated with **A** breast and **B** prostate cancer in the unidimensional (1D, purple violins) and the three-dimensional (3D, yellow violins) genome structure, and then mapped to their target genes. In each violin, the horizontal black line is the median, the narrow gray box shows the first and third quantiles, and whiskers mark the minimum and maximum values excluding outliers, which are represented by black diamonds

Next, we analyze the disease association of TFs linked to SNPs highly associated with cancer (Fig. 4) and their target genes (Fig. 5) with statistics reported in Table 2. The distribution of DA scores for TFs mapped to breast cancer is presented in Fig. 4A. Here, the median DA score of 12.0 in the 3D genome structure is higher than 3.0 in 1D. In addition, Fig. 5A shows that the median DA score of TF target genes is also higher in 3D (4.7) compared to 1D (2.7). In the absence of numerical DA scores for TFs linked to SNPs highly associated with prostate cancer, we conducted the analysis using the fraction of disease-associated TF (Fig. 4B). On average, about two-thirds of TF mapped to SNPs in 3D are disease-associated, whereas this fraction is only one-third in 1D. Further, Fig. 5B shows that the median DA score of the corresponding target genes is higher in 3D (3.2) than in 1D (1.9). In contrast to active enhancers, IQRs for TFs are similar between 1D and 3D, except for the distribution of DA scores for TF target genes in prostate cancer (1.6 and 0.7, respectively, Table 2).

### Examples of gene regulation through chromatin interactions in breast cancer

**Fig. 6:**
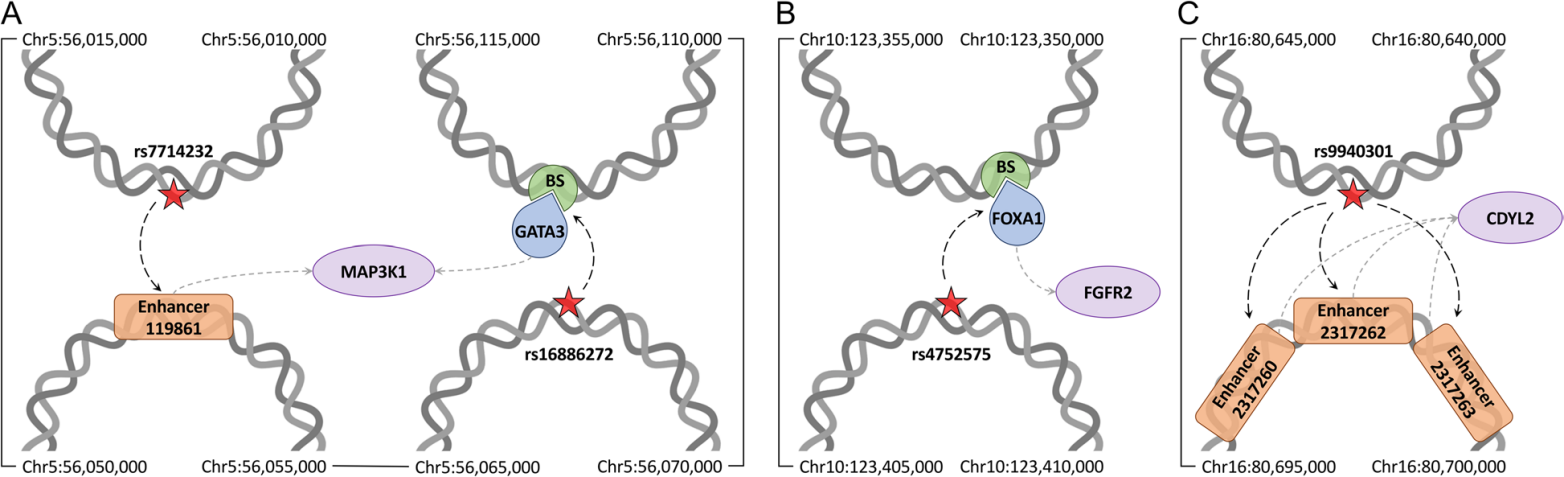
Case studies for genetic variants related to breast cancer. The possible effects of SNPs on the expression of **A** MAP3K1, **B** FGFR2, and **C** CDYL2 genes are presented in the context of the 3D genome structure. Red stars are SNPs highly associated with a disease at a *p*-value of ≤5 × 10^− 8^ affecting regulatory elements through long-range physical interactions according to dashed black arrows. Dashed gray arrows link regulatory elements, including transcription factors (blue teardrops) and their binding sites (BS, green sectors), and enhancers (orange rounded rectangles) to target genes (purple ovals). Chromatin fragments from the Hi-C data (gray double helices) annotated with their start and end coordinates are connected by solid black lines showing their order in the linear genome

We present several case studies in order to illustrate the significance of the 3D genome structure in linking genetic variation to gene regulation in breast cancer (Fig. 6). The locations of genomic elements discussed in this section and their association with breast cancer are provided in Supplementary Tables S1-S3. The first example is mitogen-activated protein kinase kinase kinase 1 (MAP3K1), a serine/threonine kinase known to play an important role in different functions of the cell [61, 62]. MAP3K1 can be activated by different stimuli, such as cytokines and growth factors, that constitute a complex signaling network controlling a diverse array of cellular functions [63]. In addition to numerous studies focused on somatic mutations in MAP3K1 [64–66], GWAS revealed associations between SNPs, including rs7714232 and rs16886272 regulating the expression of MAP3K1, and breast cancer [67, 68]. Further, multiple transcription factors, such as ER-α, FOXA1 and GATA3, were shown to upregulate the expression of MAP3K1 through long range chromatin interactions [67]. Figure 6A shows that rs7714232 at position Chr5:56,011,357 is in contact with a chromatin fragment containing an active enhancer 119,861, and rs16886272 at position Chr5:56,067,434 is in contact with a fragment containing a putative binding site for transcription factor GATA3 predicted with a *p*-value of 2.2 × 10^− 5^. Enhancer 119,861 is associated with breast cancer at a *p*-value of 2.4 × 10^− 5^ and GATA3 has a high disease association score of 5.9. MAP3K1, which itself has a high disease association score of 5.3, is a target gene for both regulatory elements. Thus, rs7714232 and rs16886272 may indirectly affect the expression of MAP3K1 through physical interactions with an active enhancer and a transcription factor binding site.

Fibroblast growth factor receptor 2 (FGFR2) belonging to the receptor tyrosine kinase family mediates the cellular signaling and plays important roles in the developmental induction, cell growth and differentiation, and cell fate [69–71]. Several studies reported the association between mutations affecting FGFR2 and breast cancer [72, 73]. For example, multiple SNPs located in the second intron of FGFR2 cause the increased expression of FGFR2 linked to cancer progression [74]. Another study reported an association between the expression of FGFR2 and the number of breast tumor initiating cells [75]. GWAS data revealed the association between FGFR2 genetic variants and the risk of breast cancer [72, 76], for instance, rs4752575 was shown to alter the expression of FGFR2 leading to the increased susceptibility to breast cancer [77]. Figure 6B shows that rs4752575 located at position Chr10:123,407,187 physically interacts with a chromatin fragment containing multiple transcription factor binding sites, including a putative binding site for forkhead box protein A1 (FOXA1) predicted with a *p*-value of 4.5 × 10^− 4^. FOXA1 is highly associated with breast cancer with a score of 5.8 and was identified as one of the master regulators of FGFR2 [78]. According to these data, we propose a new model explaining the high association of rs4752575 with breast cancer at a *p*-value of 5.5 × 10^− 9^. Specifically, rs4752575 may dysregulate the expression of FGFR2 through the chromatin interaction with the binding site for FOXA1, a master regulator of the FGFR2 gene.

Chromodomain Y-like protein 2 (CDYL2) is a putative epigenetic factor belonging to a family of proteins characterized by the presence of N-terminal chromodomain that binds methylated histones H3K9 and H3K27 [79–81]. CDYL2 has been identified as either a tumor suppressor or oncogene depending on the cancer type [82, 83]. Moreover, CDYL2 was reported to be overexpressed in breast cancer supporting its role in disease progression [84]. The transcript variants of CDYL2 were found to be differently associated with breast cancer, suggesting a new therapeutic strategy targeting specific CDYL2 isoforms [85]. Several genetic variants related to CDYL2 have been identified by GWAS to be associated with breast cancer progression and development, including rs13329835 found in the intergenic region of CDYL2 gene [86, 87]. Another variant, rs9940301, at position Chr16:80,641,906 is strongly associated with breast cancer progression in women of African ancestry with a *p*-value of 2.0 × 10^− 9^ [88]. According to the Hi-C data (Fig. 6C), rs9940301 is in contact with a chromatin fragment containing three putative enhancers, 2,317,260, 2,317,262, and 2,317,263, all associated with breast cancer with a *p*-value of 9.0 × 10^− 6^. These enhancers activate the expression of the CDYL2 gene suggesting a new association mechanism between rs9940301 and breast cancer through physical interactions with enhancers regulating CDYL2 gene expression.

### Examples of gene regulation through chromatin interactions in prostate cancer

**Fig. 7:**
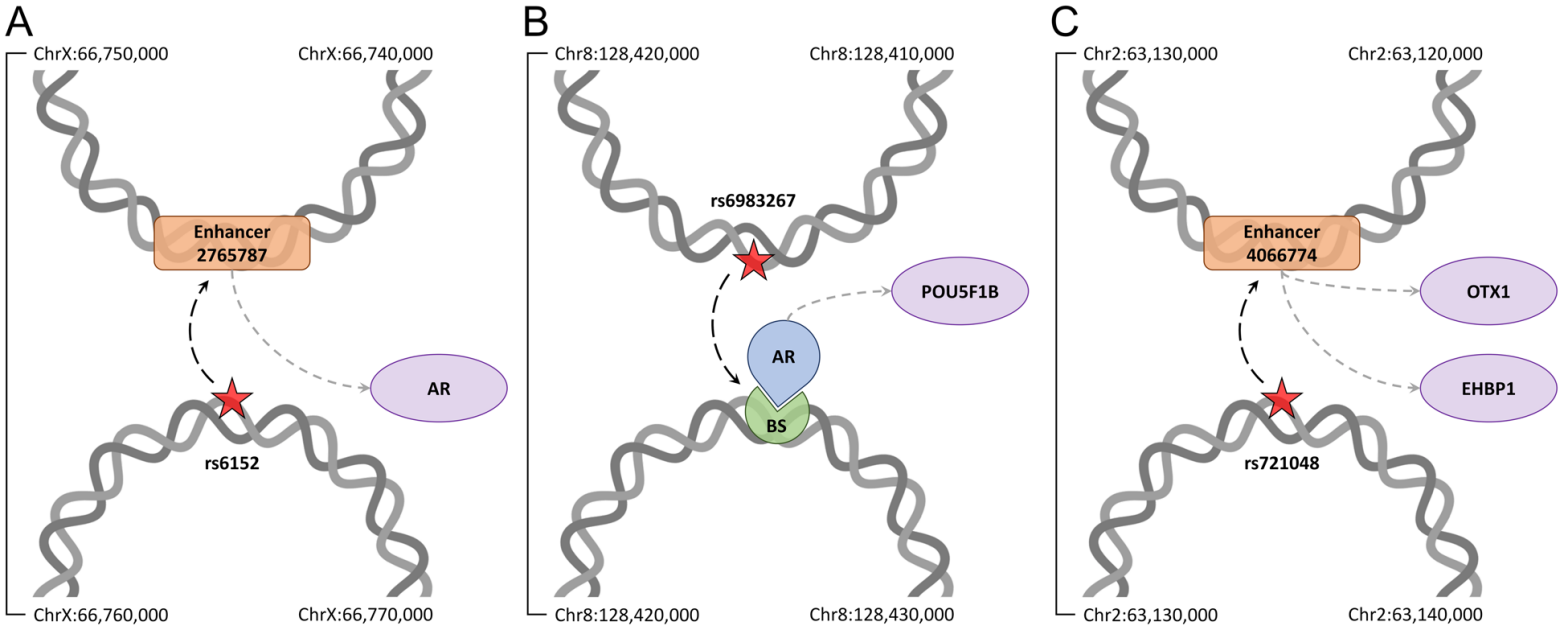
Case studies for genetic variants related to prostate cancer. The possible effects of SNPs on the expression of **A** AR, **B** POU5F1B, and **C** OXT1 and EHBP1 genes are presented in the context of the 3D genome structure. Red stars are SNPs highly associated with a disease at a *p*-value of ≤5 × 10^− 8^ affecting regulatory elements through long-range physical interactions according to dashed black arrows. Dashed gray arrows link regulatory elements, including transcription factors (blue teardrops) and their binding sites (BS, green sectors), and enhancers (orange rounded rectangles) to target genes (purple ovals). Chromatin fragments from the Hi-C data (gray double helices) annotated with their start and end coordinates are connected by solid black lines showing their order in the linear genome

Figure 7 presents selected cases demonstrating how the genetic variation in prostate cancer can be linked to gene regulation by analyzing the 3D genome structure. The locations of genomic elements discussed in this section and their association with prostate cancer are provided in Supplementary Tables S1-S3. Androgen receptor (AR) is a master regulator belonging to the nuclear receptor superfamily [89]. It acts as a transcription factor binding a specific ligand molecule to control the expression of targeted genes [90]. Prostate function primarily depends on the androgen signaling axis through the regulation of AR target genes [91]. AR is highly associated with prostate cancer with a score of 8.0, which is consistent with observations that it is often overexpressed in prostate cancer [92] and mutations in the AR gene are present in a large population of castration-resistant prostate cancer patients [93, 94]. The GWAS data revealed that numerous SNPs near the AR locus are associated with prostate cancer [95, 96]. For instance, rs6152 is located in the first exon of the AR gene and it is associated with a susceptibility to prostate cancer at a *p*-value of 1.5 × 10^− 12^ [97, 98]. We also found that rs6152 at position ChrX:66,765,627 forms a contact with a chromatin fragment containing an active enhancer 2,765,787 associated with prostate cancer at a *p*-value of 1.6 × 10^− 2^ and affecting the expression of AR (Fig. 7A). Thus, the physical interaction between rs6152 and an enhancer may play a role in the regulation of AR gene expression during prostate cancer progression.

Octamer-binding transcription factor 4 (OCT4), a member of the POU domain-containing family of transcription factors, is expressed in embryonic and adult stem cells [99]. Although six different pseudogenes identified for OCT4 are not expressed, these elements are believed to play a role in the regulation of OCT4 expression [100, 101]. Interestingly, two of these OCT4 pseudogenes, POU5F1P5 and POU5F1B, were found to be transcribed in cancer cells [102]. POU5F1B was shown to be overexpressed in gastric cancer and its knockdown confirmed a role for POU5F1B in the promotion of tumor cell growth [103]. Further, the methylation level near the POU5F1B gene [104] and the genetic variation around that region [105] were found to be associated with the prostate cancer risk. For instance, rs6983267 was reported to be in linkage disequilibrium with the open-reading frame of the POU5F1B gene among people of European ancestry and associated with the expression of POU5F1B in prostate of white subjects [106]. Located at position Chr8:128,413,305, rs6983267 is associated with prostate cancer at a *p*-value of 2.8 × 10^− 141^. Figure 7B shows that it is also in contact with a chromatin fragment containing multiple putative transcription factor binding sites including a confidently predicted binding site for AR with a *p*-value of 3.5 × 10^− 4^, which regulates the expression of POU5F1B. According to these findings, rs6983267 may play a role in regulating PO5F1B expression by affecting the binding of AR to its binding site.

EH domain-binding protein 1 (EHBP1) gene encodes Eps15 homology domain binding protein playing a role in endocytic trafficking [107]. Recently, GWAS reported the association of a genetic variant rs721048, located within one of the introns of the EHBP1 gene, and the susceptibility to prostate cancer [28, 108] with a *p*-value of 5.0 × 10^− 22^. Interestingly, Fig. 7C shows that rs721048 at position Chr2:63,131,731 forms a contact with a chromatin fragment containing an active enhancer 406,774 associated with prostate cancer at a *p*-value of 1.5 × 10^− 4^ that regulates the expression of EHBP1. Further, enhancer 406,774 also regulates the expression of orthodenticle homeobox 1 (OTX1), a transcription factor playing a critical role in multiple developmental processes, such as the neuronal differentiation [109]. Several studies reported the hypermethylation of the OTX1 gene promoter region in non-small lung cancer [110, 111] and an altered OTX1 expression in medulloblastoma and other brain tumors [112]. It is important to note that the expression of OTX1 is also associated with prostate cancer risk [113]. Further, OTX1 is one of several transcription factors involved in tumor-specific enhancer networks and it was found to be linked to active enhancers in prostate adenocarcinoma [114]. Our data suggest that rs721048 may be associated with prostate cancer through the disruption of the mechanism of action of certain tumor-specific enhancers causing the dysregulation of the expression of OTX1 and EHBP1 genes.

### Mapping genetic variants to topologically associating domains

**Fig. 8:**
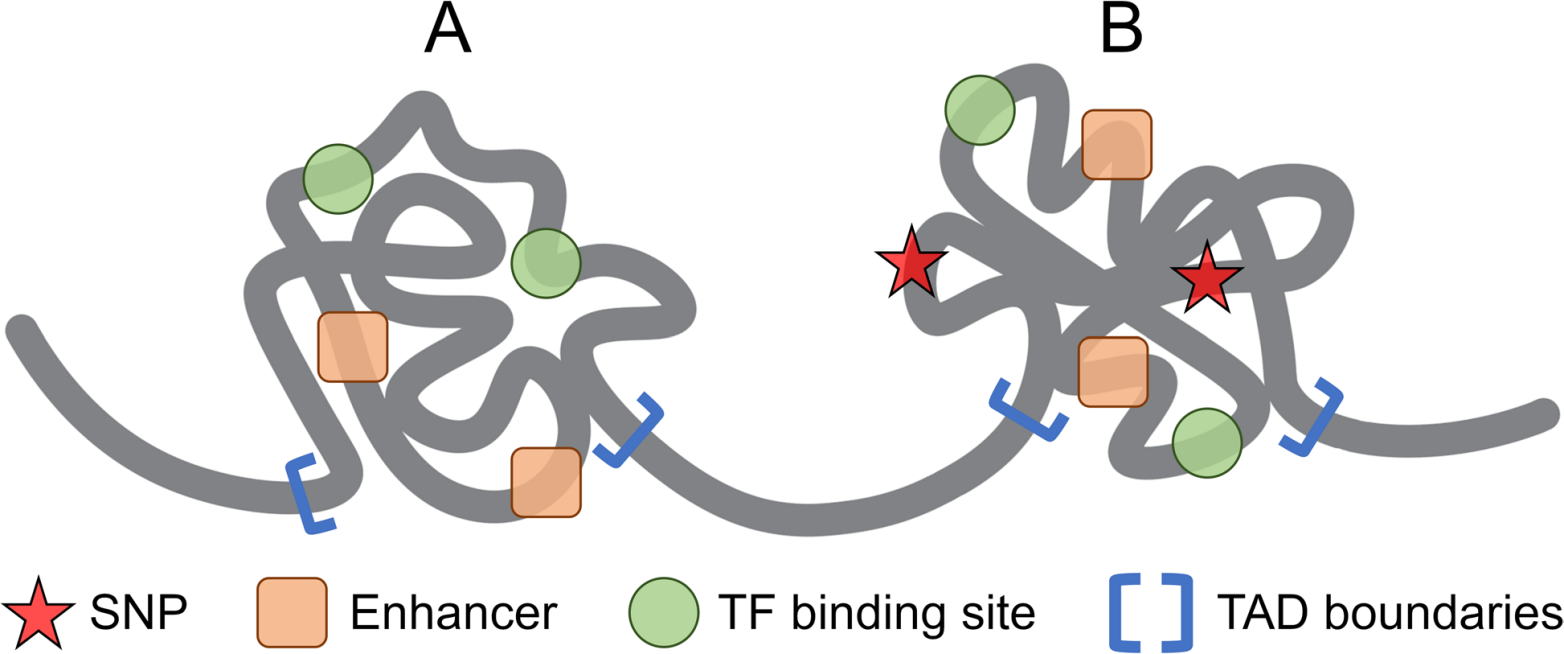
Schematic representation of topologically associating domains (TADs). Red stars are SNPs highly associated with a disease at a *p*-value of ≤5 × 10^− 8^, orange rounded squares represent enhancers, green circles represent transcription factors, and blue square brackets show the location of TAD boundaries. TADs are divided into two groups, **A** control TADs containing no highly associated SNPs and **B** SNP-rich TADs carrying at least one highly associated variant

Next, TADs were identified from the Hi-C data and all regulatory elements and SNPs were mapped to these domains as shown in Fig. 8. Based on the presence of SNPs highly associated with cancer, the resulting TADs are divided into two groups, TADs containing no SNPs (control, Fig. 8A) and TADs containing at least one SNP with a *p*-value of ≤5 × 10^− 8^ according to the GWAS data (SNP-rich, Fig. 8B). Specifically, we identified the total of 21,157 TADs from the Hi-C data for breast cancer, including 30 TADs enriched with disease-associated SNPs. Among 30 SNP-rich TADs, 26 also contain enhancers (477 in total) and 17 contain TF binding sites (36 in total). The control dataset for breast cancer comprises 16,473 TADs containing 259,780 enhancers and 10,463 TADs containing 23,890 binding sites for TFs. In addition, the total of 17,435 TADs were detected from the Hi-C data for prostate cancer, including 304 TADs enriched with disease-associated SNPs; 291 of these SNP-rich TADs contain enhancers (7940 in total) and 241 contain TF binding sites (686 in total). The control dataset for prostate cancer comprises 13,587 TADs containing 250,789 enhancers and 10,070 TADs containing 22,562 binding sites for TFs.

We first take a glance at selected genetic variants highly associated with breast (4 SNPs) and prostate (3 SNPs) cancers discussed above in order to determine whether gene regulatory elements located in their spatial proximity according to the Hi-C data reside in the same TAD. Interestingly, this holds true in almost all cases. Both variants rs7714232 and rs16886272 associated with breast cancer, enhancer 119,861, and a binding site for transcription factor GATA3 are located in TAD 6450 whose boundaries are Chr5:56,010,000 – Chr5:56,140,000. Further, variant rs9940301 along with all three enhancers, 2,317,260, 2,317,262, and 2,317,263, reside in the same TAD 17447 (Chr16:80,630,000 – Chr16:80,950,000). Variant rs4752575 and FOXA1 binding site belong to neighboring TADs 12,498 (Chr10:123,360,000 – Chr10:123,460,000) and 12,497 (Chr10:123,330,000 – Chr10:123,360,000), respectively. In prostate cancer, TAD 1830 (Chr2:63,010,000 – Chr2:63,160,000) contains both rs721048 and enhancer 406,774, TAD 8618 (Chr8:128,410,000 – Chr8:128,490,000) contains both rs6983267 and AR binding site, and TAD 16953 (ChrX:66,530,000 – ChrX:67,270,000) contains both rs6152 and enhancer 2,765,787. On that account, we expect TADs enriched in disease-associated SNPs to also contain those gene regulatory elements having higher disease association compared to control TADs, which is investigated in the following section.

### Association of SNPs and regulatory elements with cancer in the context of TADs

**Fig. 9:**
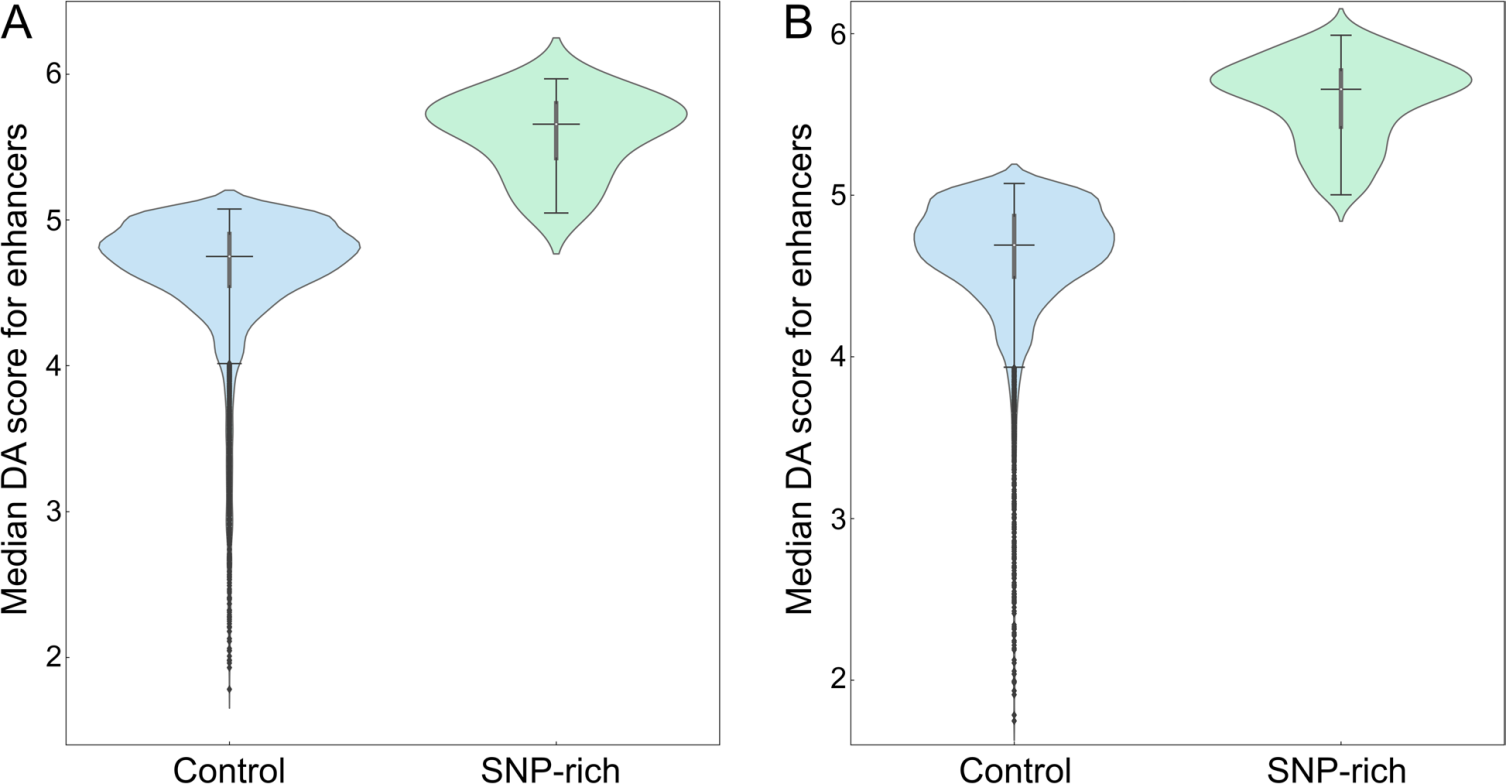
Distribution of disease-association (DA) scores for enhancers within TADs. DA scores against **A** breast and **B** prostate cancer are calculated for enhancers present in control (blue violins) and SNP-rich (green violins) TADs. In each violin, the horizontal black line is the median, the narrow gray box shows the first and third quantiles, and whiskers mark the minimum and maximum values excluding outliers, which are represented by black diamonds

**Fig. 10:**
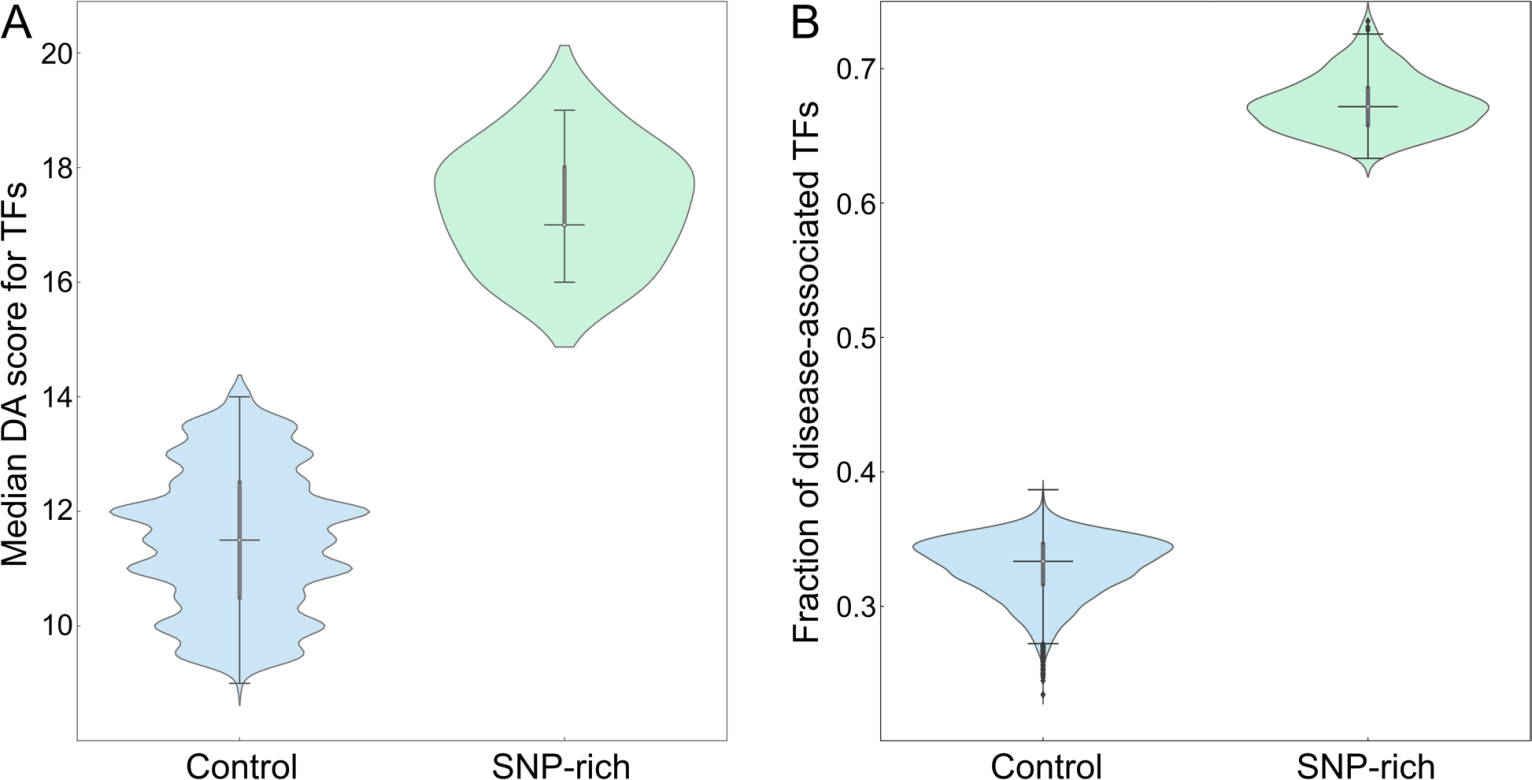
Analysis of the disease association of transcription factors (TFs) within TADs. **A** The distribution of disease-association (DA) scores against breast cancer and **B** the fraction of TFs associated with prostate cancer. Blue violins show the distribution of DA scores and the fraction within control TADs, whereas green violins show the distribution within SNP-rich TADs. In each violin, the horizontal black line is the median, the narrow gray box shows the first and third quantiles, and whiskers mark the minimum and maximum values excluding outliers, which are represented by black diamonds

In order to verify the assertion that those TADs carrying genetic variants highly associated with cancer also contain gene regulatory elements whose disease association is high, we first calculated DA scores for enhancers located within control and SNP-rich TADs. The distribution of DA scores is shown in Fig. 9 with the corresponding statistics reported in Table 3. Compared to a median DA score of 4.75 for control TADs in breast cancer, SNP-rich TADs contain enhancers with a higher median DA score of 5.66 (Fig. 9A). A median DA score of 5.66 for enhancers located in SNP-rich TADs is higher than a value of 4.69 for those belonging to control TADs in prostate cancer as well (Fig. 9B). Similar to enhancers, Fig. 10 shows the distribution of DA scores computed for TFs residing in SNP-rich and control TADs with the corresponding statistics reported in Table 3. In breast cancer, the median DA score of 11.5 for TFs located in control TADs is lower than a value of 17.0 for those present in SNP-rich TADs, whereas in prostate cancer, the fraction of disease-associated TFs in SNP-rich TADs is twice as high as in control TADs (Fig. 10B). Further, Table 3 shows that IQRs for enhancers and TFs are very similar between SNP-rich and control TADs in both cancers. These results demonstrate that disease-associated genetic variants and gene regulatory elements indeed tend co-localize within certain TADs identified based on the 3D genome structure.

**Table 3:** Disease association (DA) statistics for enhancers and transcription factors (TFs) within TADs in breast and prostate cancer. TADs are divided into two groups, containing no SNPs with a significant association to disease (control) and those containing at least one SNP with a significant disease association (SNP-rich). Statistics include the number of TADs used in the analysis, quantiles, and the inter-quantile range (IQR)

Statistic	Breast cancer				Prostate cancer			
	Enhancer DA score		TF DA score		Enhancer DA score		Fraction of DA-TFs^**a**^	
	Control	SNP-rich	Control	SNP-rich	Control	SNP-rich	Control	SNP-rich
# of TADs	16,473	26	10,463	17	13,587	291	10,070	241
1^st^ quantile	4.55	5.42	10.5	17.0	4.50	5.42	0.32	0.66
2^nd^ quantile	4.75	5.66	11.5	17.0	4.69	5.66	0.33	0.67
3^rd^ quantile	4.90	5.80	12.5	18.0	4.87	5.77	0.35	0.69
IQR	0.35	0.38	2.0	1.0	0.37	0.35	0.03	0.03

^a^ Fraction of disease-associated TFs within TADs

## Discussion

Although a large number of genetic variants associated with cancer have been identified by GWAS, the exact mechanisms by which these mutations affect the phenotype have not yet been fully elucidated. A significant challenge in explaining the functional mechanisms of SNPs in cancer initiation and progression arises from the fact that the vast majority of disease-associated variants are located within the non-coding regions of the genome. Non-coding SNPs are thought to exert their pathological effects by altering gene regulation rather than directly affecting the sequence, structure, and function of gene products. Accumulated data on the 3D genome structure collected from Hi-C experiments offer a unique opportunity to investigate the effects of genetic variation, particularly those located in the non-coding complement of the human genome, on gene regulation leading to cancer pathophenotypes. In this communication, we integrated the large-scale data provided by GWAS with the information on chromatin structure and interactions in breast and prostate cancers in order to systematically evaluate the effects of SNPs on gene regulatory mechanisms. We are specifically interested in the comparison of this 3D method utilizing the Hi-C data to a 1D proximity approach assuming that genetic variants affect regulatory elements located down- and up-stream in the linear DNA.

Focusing on SNPs highly associated with breast and prostate cancers, we conducted a comprehensive analysis of their relationships to various enhancers and binding sites for transcription factors regulating the expression of their target genes. Considering a vast body of evidence supporting the association of many regulatory elements with cancer phenotypes, we propose that genetic variants forming physical contacts with these elements may contribute to the development and progression of disease by altering the expression levels of cancer-related genes. There are several benefits of including the Hi-C data in the analysis of the effects of genetic variation on gene expression. The 3D genome structure allows for a more efficient mapping between variants and regulatory elements compared to the unidimensional genome lacking physical chromatin interactions [115, 116]. Importantly, the disease association scores of enhancers and transcription factors mapped to cancer-associated SNPs through chromatin contacts are consistently higher compared to those identified using a linear DNA model. This also holds true for the target genes of these regulatory elements.

Our results are in line with numerous studies demonstrating that the 3D structure of the genome, which facilitates certain DNA-DNA interactions regulating gene expression [117], can effectively be used to reveal the functional mechanisms of genetic variation as well as candidate genes related to cancer [118]. For instance, potentially functional non-coding mutations were identified by integrating cancer genome variation with cis-regulatory networks, long-range chromatin interactions, and transcriptomic data [119]. This study demonstrated that frequently mutated regulatory elements not only show long-range chromatin interactions and mRNA abundance associations with target genes, but also are enriched in motif-rewiring mutations and structural variants. Another research characterized the mutational landscape of gene-regulatory and chromatin architectural elements in whole cancer genomes with transcriptional and pathway activity, functional conservation and recurrent driver events [120]. A statistical model to quantify mutational enrichment or depletion in classes of genomic elements through megabase-scale effects revealed that an increased mutation frequency in transcription start sites associates with mRNA abundance in most cancer types, while open-chromatin regions are generally enriched in mutations.

We finally investigated the relationship among genetic variants, regulatory elements, and their target genes in the context of TADs, relatively small, compact, and self-interacting genomic regions [121]. As anticipated, we found that those TADs carrying cancer-associated SNPs also contain enhancers and binding sites for transcription factors whose disease association is generally higher compared to regulatory elements located in control TADs devoid of high-risk SNPs. This analysis can further be expanded to include genome-wide epigenome patterns highlighting an important role of the DNA methylation in the maintenance of 3D genome regulation. Interestingly, DNA methylation was found to intrinsically modulate chromatin structure and function by increasing chromatin condensation in peri-centromeric regions, decreasing the overall DNA flexibility, and favoring the heterochromatin state [122]. Further, it has been shown that cancer-related methylation loss is associated with the deregulation of 3D genome organization leading to the disruption of the genome compartmentalization [123]. DNA methylation can also inactivate TAD boundaries leading to the concomitant activation of key cancer drivers by enhancers located outside their normal TADs through long-range chromatin interactions [124].

Integrating the chromatin structure with the multi-omics data is a promising approach to study how the spatial organization of the genome affects gene regulation through physical interactions between various genomic regions. This technique also holds promise to not only reveal hot spots within the human genome linked to disease, but also investigate subtle differences between the genetic makeup of individuals leading to varying levels of disease risk and progression. Overall, our work provides a new perspective for investigating the effects of genetic variation on gene regulation in cancer through the large-scale analysis of long-range chromatin interactions shaping the 3D genome structure.

## Methods

### Hi-C data

Hi-C data at 5 kbp resolution collected for the human mammary epithelial cell line were downloaded from the Gene Expression Omnibus database (GEO accession: GSE63525) [50]. The Hi-C data at 10 kbp resolution collected for the human normal prostate cell line were downloaded from the Gene Expression Omnibus database (GEO accession: GSM3564252) [125]. In order to effectively detect significant chromatin contacts, statistical confidence estimates for Hi-C contact maps were computed with the Fit-Hi-C programming application [126]. Specifically, the latest reimplementation, FitHiC2 v2.0.7, was used to perform the genome-wide analysis of the high-resolution Hi-C data for breast and prostate cancers, including intra- and inter-chromosomal contacts. This software first computes binomial *p*-values for the significance of observing a contact count that is at least equal to the observed integer count value or higher. *P*-values are then subjected to multiple testing correction using the Benjamini-Hochberg procedure to obtain *q*-values representing the minimum false discovery rate (FDR) threshold at which the contact is considered significant [127]. We excluded low-confidence interactions keeping only those contacts whose *q*-values are ≤0.05 [128].

### Genome-wide association studies

In this study, we used the GWAS data for breast cancer generated for 122,977 cases and 105,974 controls of European ancestry and 14,068 cases and 13,104 of East Asian ancestry [129], and for prostate cancer generated for 46,939 cases and 27,910 controls of European ancestry [130]. We first identified SNPs having OncoArray accession numbers resulting in 568,712 SNPs for breast cancer and 13,502,794 SNPs for prostate cancer, and then we selected 808 (breast cancer) and 13,447 (prostate cancer) highly associated SNPs with a *p*-value of ≤5 × 10^− 8^ according to the published work [131].

### Gene regulatory elements

Data on enhancers, including their genomic location, target genes, and disease association scores were obtained from the Human Enhancer Disease Database (HEDD) database [132]. HEDD provides comprehensive information for about 2.8 million human enhancers identified by ENCODE, FANTOM5 and RoadMap with disease association scores based on enhancer-gene and gene-disease associations. In this study, we used 262,490 enhancers related to breast cancer and 267,453 enhancers related to prostate cancer. The data on transcription factors were obtained from the TF2DNA database containing 1306 TFs, 19,190 target genes, and 24,634,759 binding sites [133]. From these data, 164 TFs associated with breast cancer were selected based on an experimental and computational pipeline integrating AccessTF, a Bayesian network model to accurately predict protein-bound DNA sequence motifs based on chromatin accessibility, with TFScore, a scoring system that rank-orders transcription factors as candidates for being important for a biological process [134]. Further, we identified 612 TFs associated with prostate cancer based on RegNetDriver, a novel computational method to identify tumorigenic drivers from the effects of coding and non-coding single nucleotide variants, structural variants, and DNA methylation changes in the DNase I hypersensitivity based regulatory network [135]. Disease association scores for enhancer and TF target genes were collected from the DISEASES database [136]. This resource provides evidence on disease-gene associations primarily computed by an automatic text mining of biomedical abstracts. A scoring scheme employed by DISEASES also integrates other types of evidence including manually curated disease-gene associations, cancer mutation data, and genome-wide association studies from multiple databases. We identified the total of 12,846 genes having an association score to breast cancer and 10,032 genes having an association score to prostate cancer.

### Genome-wide mapping of genetic variants to regulatory elements

In the unidimensional approach, SNPs highly associated with breast and prostate cancer were mapped to enhancers and TF binding sites in the linear proximity using a 5 kbp window (2.5 kbp up and 2.5 kbp downstream from the SNP) for breast cancer and a 10 kbp window (5 kbp up and 5 kbp downstream from the SNP) for prostate cancer. These window sizes were selected to match the resolution of the Hi-C data. For each SNP, we then calculated the median association score of mapped enhancers and TFs as well as their target genes with the exception of TFs in prostate cancer, for which we computed the fraction of disease-associated TFs. In the three-dimensional approach, each highly associated SNP was mapped to regulatory elements present in a chromatin fragment forming the most confident contacts with the lowest *q*-value computed by the FitHiC2 software. When multiple fragments have the same lowest *q*-value, we selected the longest-range interaction. This way, the same number of chromatin fragments are utilized by both 1D and 3D approaches. The median association scores of regulatory elements and their target genes mapped through physical interactions were calculated in a similar manner as in the 1D analysis.

### Topologically associating domains

TADs were identified from intra-chromosomal contacts for each chromosome with TopDom v0.10.0 [137]. For each TAD, we calculated the median association scores for regulatory elements present in that domain as well as for their target genes. TADs were then divided into two groups, SNP-rich carrying at least one genetic variant highly associated with cancer at a *p*-value of ≤5 × 10^− 8^ and control TADs containing no highly associated SNPs.

## Supplementary Information

**Additional file 1 **: **Table S1**. Genetic variants selected to illustrate the significance of the 3D genome structure in gene regulation in breast cancer. **Table S2**. Enhancers forming physical contacts with genetic variants listed in Table S1 according to the 3D genome structure. **Table S3**. Transcription factor (TF) binding sites in physical contact with genetic variants listed in Table S1 according to the 3D genome structure.

## Data Availability

The generated data is available through the Open Science Framework (https://osf.io/, accession 4xwc6).
