# Supplementary material for: Exploring the effects of genetic variation on gene regulation in cancer in the context of 3D genome structure
Source: BMC Genom Data. 2022 Feb 17;23:13. doi: 10.1186/s12863-021-01021-x (PMC8851830; doi:10.1186/s12863-021-01021-x)
Supplement: Supplementary file 1 — Additional file 1 : Table S1. Genetic variants selected to illustrate the significance of the 3D genome structure in gene regulation in breast cancer. Table S2. Enhancers forming physical contacts with genetic variants listed in Table S1 according to the 3D genome structure. Table S3. Transcription factor (TF) binding sites in physical contact with genetic variants listed in Table S1 according to the 3D genome structure. [file 12863_2021_1021_MOESM1_ESM.pdf]

**Supplementary Information**

**Table S1.** Genetic variants selected to illustrate the significance of the 3D genome structure in gene regulation in breast cancer.

| Genetic variant | Genomic position | TAD[a] | Disease association[b] |
|---|---|---|---|
| | *Breast cancer* | | |
| rs7714232 | Chr5:56,011,357 | 6450 | $9.1\times10^{-13}$ |
| rs16886272 | Chr5:56,067,434 | | $4.7\times10^{-9}$ |
| rs4752575 | Chr10:123,407,187 | 12498 | $5.5\times10^{-9}$ |
| rs9940301 | Chr16:80,641,906 | 17447 | $2.0\times10^{-9}$ |
| | *Prostate cancer* | | |
| rs6152 | ChrX:66,765,627 | 16953 | $1.5\times10^{-12}$ |
| rs6983267 | Chr8:128,413,305 | 8618 | $2.8\times10^{-141}$ |
| rs721048 | Chr2:63,131,731 | 1830 | $5.0\times10^{-22}$ |

[a] ID of topologically associating domain, [b] *p*-value from genome-wide association studies.

**Table S2.** Enhancers forming physical contacts with genetic variants listed in Table S1 according to the 3D genome structure.

| Enhancer ID | Genomic position | TAD[a] | Disease association[b] | Target genes | DA-score[c] |
|---|---|---|---|---|---|
| *Breast cancer* | | | | | |
| 119861 | Chr5:56,052,902-56,053,241 | 6450 | $2.5\times10^{-5}$ | MAP3K1 | 5.3 |
| 2317260 | Chr16:80,698,090-80,698,168 | | | | |
| 2317262 | Chr16:80,698,782-80,698,981 | 17447 | $9.0\times10^{-6}$ | CDYL2 | 2.0 |
| 2317263 | Chr16:80,699,105-80,699,234 | | | | |
| *Prostate cancer* | | | | | |
| 2765787 | ChrX:66,744,392-66,744,590 | 16953 | $1.6\times10^{-2}$ | AR | 8.0 |
| 406774 | Chr2:63,122,149-63,122,353 | 1830 | $1.5\times10^{-4}$ | OTX1 | 1.3 |
| | | | | EHBP1 | 3.2 |

[a] ID of topologically associating domain, [b] *p*-value from the HEDD database, [c] disease association score from the DISEASES database.

**Table S3.** Transcription factor (TF) binding sites in physical contact with genetic variants listed in Table S1 according to the 3D genome structure.

| TF | TF DA-score[a] | Genomic position | TAD[b] | TF binding score[c] | Target genes | TG DA-score[a] |
|---|---|---|---|---|---|---|
| | | | *Breast cancer* | | | |
| GATA3 | 5.9 | Chr5:56,109,399-56,111,399 | 6450 | 1090 ($2.2\times10^{-5}$) | MAP3K1 | 5.3 |
| FOXA1 | 5.8 | Chr10:123,353,272-123,355,272 | 12497 | 959 ($4.5\times10^{-4}$) | FGFR2 | 5.6 |
| | | | *Prostate cancer* | | | |
| AR | 8.0 | Chr8:128,426,356-128,428,356 | 8618 | 1005 ($3.5\times10^{-4}$) | POU5F1B | 3.7 |

[a] disease association score from the DISEASES database, [b] ID of topologically associating domain, [c] TF binding score (*p*-value) from the TF2DNA database.
